# Effects of Trophic Level and Metamorphosis on Discrimination of Hydrogen Isotopes in a Plant-Herbivore System

**DOI:** 10.1371/journal.pone.0032744

**Published:** 2012-03-28

**Authors:** Jacob M. Peters, Nathan Wolf, Craig A. Stricker, Timothy R. Collier, Carlos Martínez del Rio

**Affiliations:** 1 Department of Zoology and Physiology, University of Wyoming, Laramie, Wyoming, United States of America; 2 U. S. Geological Survey, Fort Collins Science Center, Denver Federal Center, Denver, Colorado, United States of America; 3 Department of Renewable Resources, University of Wyoming, Laramie, Wyoming, United States of America; University of California, Berkeley, United States of America

## Abstract

The use of stable isotopes in ecological studies requires that we know the magnitude of discrimination factors between consumer and element sources. The causes of variation in discrimination factors for carbon and nitrogen have been relatively well studied. In contrast, the discrimination factors for hydrogen have rarely been measured. We grew cabbage looper caterpillars (*Trichoplusia ni*) on cabbage (*Brassica oleracea*) plants irrigated with four treatments of deuterium-enriched water (δD = −131, −88, −48, and −2‰, respectively), allowing some of them to reach adulthood as moths. Tissue δD values of plants, caterpillars, and moths were linearly correlated with the isotopic composition of irrigation water. However, the slope of these relationships was less than 1, and hence, discrimination factors depended on the δD value of irrigation water. We hypothesize that this dependence is an artifact of growing plants in an environment with a common atmospheric δD value. Both caterpillars and moths were significantly enriched in deuterium relative to plants by ∼45‰ and 23‰ respectively, but the moths had lower tissue to plant discrimination factors than did the caterpillars. If the trophic enrichment documented here is universal, δD values must be accounted for in geographic assignment studies. The isotopic value of carbon was transferred more or less faithfully across trophic levels, but δ^15^N values increased from plants to insects and we observed significant non-trophic ^15^N enrichment in the metamorphosis from larvae to adult.

## Introduction

The use of stable isotopes in ecology often demands that we know how faithfully the isotopic composition of resources is incorporated into the tissues of consumers [1], [2]. The metric most often used to estimate this faithfulness is called a trophic discrimination factor and is defined as the difference in the delta value of the consumer's tissues and that of its diet (ie. ΔX_consumer-diet_ = δX_consumer_−δX_diet_) [2]. For carbon and nitrogen isotopes, trophic discrimination factors [3], [4] and the mechanisms that determine the magnitude of discrimination factors are relatively well understood (reviewed by [1]). Despite the frequency with which hydrogen isotopes are used to study various aspects of animal ecology (reviewed by [1]) we lack a mechanistic understanding of both the incorporation and consequent trophic discrimination of hydrogen isotopes between resources and consumer tissues [5], [6]. This lack of understanding poses complications for studies using hydrogen isotope analysis to study animal movements [7]. With this technique ecologists attempt to match the δD signature of an animal's tissue (typically feathers or hair) with the deuterium signature of local precipitation in order to approximate the geographic location at which the tissue was grown [7]. Although previous studies have highlighted the potential utility of this method (reviewed by [7]), the accuracy of geographical assignments based on deuterium signatures is limited without reliable estimates of the discrimination among hydrogen isotopes between consumer tissues and the consumer's resources.

The hydrogen in consumer tissues can either be derived from diet or ingested water [8]. Differences in the δD value of these two sources may complicate the calculation of an accurate precipitation to tissue discrimination factor, thereby leading to incorrect geographic assignment of consumers [9], [10]. Consequently, it is important to understand how faithfully the δD value of precipitation is reflected in dietary resources such as plant matter and insects. We documented changes in δD values of both plants and consumer tissues as a function of the δD value of source water in a simple food web: a plant and a monophagous herbivore that relies on the plant for both food and pre-formed water. We also designed our experiment to estimate the discrimination factors between plants, caterpillars, and moths for ^13^C and ^15^N. Specifically, we examined the hypothesis, proposed by Tibbets et al. (2007) [11], that metamorphosis can cause enrichment of ^15^N in the tissues of adult insects relative to that of their larvae.

## Methods

We manufactured irrigation water by adding 99.8% deuterium oxide (D_2_O) (Cambridge Isotope Laboratories, Inc. Andover MA) to tap water to yield 4 water treatments (δD = −131, −88, −48, and −2‰, respectively). Because water autionizes, D atoms from the D_2_O used to spike irrigation water become randomly distributed among the molecules of tap water. These treatments roughly span the range of δD values in continental rainwater [12]. We planted 6 cabbage plants per treatment (*Brassica oleracea*) in 0.3×0.3 m pots filled with 2∶1 potting soil (Metro-mix 900, Sun Gro Horticulture) to sand mixture. In order to prevent evaporation from the surface of the soil, we covered the soil with thin plastic wrap and watered four groups of plants with the four respective water treatments every 3 days (≈0.5 L). We maintained the plants at a natural photoperiod, from 11/16/2009 to 02/05/2010, in a greenhouse with daytime and nighttime temperatures of 24°C and 22°C, respectively. We placed approximately 15 cabbage looper eggs (*Trichoplusia ni*), purchased from Benzon Research, Inc., on the underside of a single leaf of each plant on 02/05/2010. In order to prevent the loopers from moving from plant to plant, we covered the plants with 0.3×0.3×0.3 ft. mesh cages (Bugdorm-1; Megaview Science Education Services Co., Ltd; Taiwan). We collected one to six 5^th^ instar larvae (0.151±0.08 g, N = 99) from each plant in glass vials. We held the larvae in vials for sufficient time to allow complete evacuation of gut contents, euthanized them by freezing, and stored them at −30°C. All remaining larvae pupated and emerged as adults. As soon as we detected the emerging adults, we collected them in glass vials and stored them at −30°C. We harvested approximately half of a leaf per cabbage plant.

We extracted water from soil, plant, larvae, and moth samples by cryogenic distillation [13] with an extraction efficiency of 97.8% (±2.4% SE, N = 61). We then dried organic samples at 40°C to constant mass, ground them, extracted lipids with petroleum ether, and dried them again. We loaded and crimp-sealed the samples (*ca.* 0.5 mg) in 3.5×5 mm tin capsules for carbon (δ^13^C), nitrogen (δ^15^N), and silver capsules for hydrogen (δD) isotope analyses [14]. Nitrogen and carbon isotope analyses were performed using a Thermo Finnigan Delta plus XP (Waltham, MA, USA) mass spectrometer operated in continuous flow mode at the University of Wyoming Stable Isotope Facility. Peptone (δ^15^N = 5.6‰, AIR, USGS40 8542) and glycine (δ^15^N = 0.7‰, AIR, IAEAN2) internal standards were used for δ^15^N and sample precision was ±0.2‰. Hydrogen isotope composition of organic matter samples was measured by virtual equilibration following Wassenaar and Hobson (2003) [14]. Hydrogen samples were pyrolyzed at 1425°C using a high temperature elemental analyzer (Thermo-Finnigan TC/EA) interfaced to an isotope ratio mass spectrometer (Thermo-Finnigan DeltaPlus XL) operated in continuous flow mode. We determined non-exchangeable δD values by normalizing isotopic data to V-SMOW using in-house lab standards (LA bear hair, −78‰, AK bear hair, −172‰) calibrated to BWB-CHS-CFS [14]. We analyzed additional in-house standards (Chitin (B2160, Elemental Microanalysis Limited), Peach Leaves (NIST 1547), Mussel tissue (NIST 2976)), and reagent-grade keratin) for quality control purposes; analytical error and accuracy were ±2‰, and precision was ±4‰. Several of the samples were outside of our calibration range, but by less than 20‰, which does not present serious extrapolation errors [15]. Hydrogen exchangeability in matrices other than keratin has received comparatively less study, however the magnitude of exchange appears to be similar [16], [17]. Despite the matrix differences, we normalized plant and invertebrate samples to keratin standards [18], [19]. We expressed all stable isotope values in delta (δ) notation 
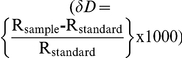
, where X is an isotope, δ is in parts per thousand (‰) deviation relative to a standard (monitoring) gas, and R_sample_ and R_standard_ are the ratios of the heavy to the light isotopes for sample and standard, respectively.

### Statistical Analysis

We used linear models to describe the relationship between the δD values of organisms and those of their hydrogen sources. These models included δD_source_ and “stage” (defined as plant, caterpillar and imago) and their interaction as independent variables and theδD of organics or body water as dependent variables. If the interaction terms in these models were statistically significant, we used Tukey's test to compare among slopes [20]. If the interaction terms were not statistically significant, we removed them from the models and estimated common slopes. We estimated discrimination factors as ΔX_tissues-source_ = δX_tissues_−δX_source_, where X is an isotope, and tested whether these discrimination factors differed from 0 using a one sample t test. We report isotope values and estimated slope and intercept values with associated standard deviations (SD) or standard errors (SE).

## Results

### Hydrogen in organic compounds

**Figure 1:**
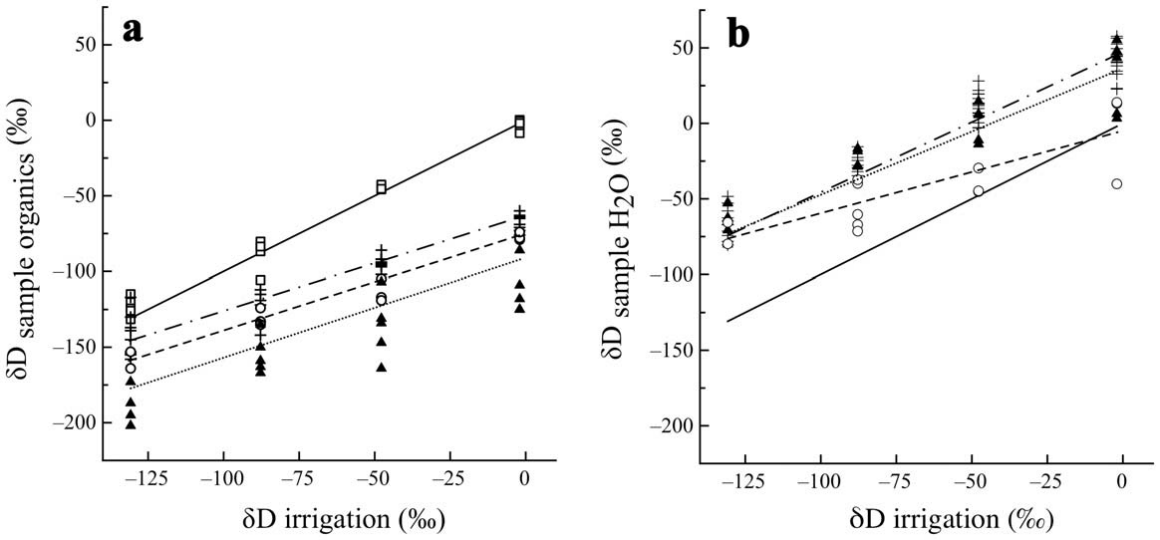
δD of organic matter and extracted water of plant and insect tissues are linearly correlated to δD of irrigation water. (a) The δD values of the organic matter of plants (solid triangles, dotted line), caterpillars (plus ‘+’ signs, barred-dotted line), and moths (open circles, barred line) increased linearly with the δD value of irrigation water (y_plant_ = 0.59x−107.5, r^2^ = 0.74; y_caterpillar_ = 0.59x−81.2, r^2^ = 0.90; y_moth_ = 0.59x−67.8, r^2^ = 0.90). These relationships had a common slope that was significantly less than 1. Note that the δD value of soil (open squares) did not differ from that of irrigation water (y = x, solid line). Plant, caterpillar, and moth tissues are significantly depleted in deuterium relative to irrigation water. (b) The δD value of water extracted from plants (solid triangles, dotted line), caterpillars (plus ‘+’ signs, barred-dotted line), and moths (open circles, barred line) was also linearly related to that of irrigation water (y_plants H2O_ = 0.73x+34.7, r^2^ = 0.86; y_caterpillar H2O_ = 0.85x+46.8, r^2^ = 0.94; y_moth H2O_ = 0.41x−19.2, r^2^ = 0.44). Extracted water samples from plants, caterpillars and moths were enriched in deuterium relative to irrigation water. The slope of the relationships of the δD value of irrigation water to water extracted from plants and caterpillars did not differ significantly. The slope of the relationship between the δD value of irrigation and that of water extracted from moths was significantly shallower.

**Figure 2:**
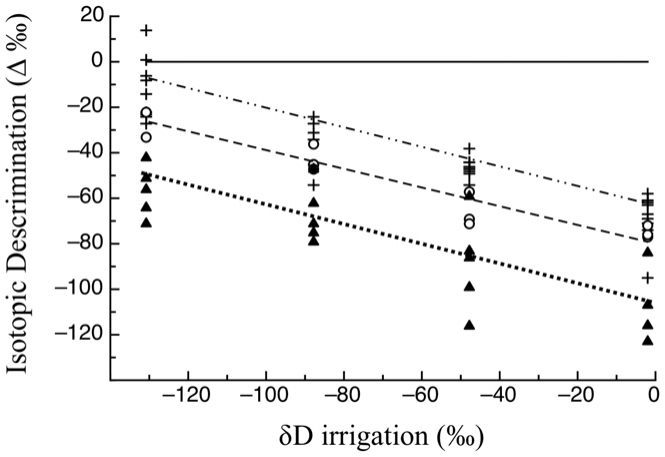
Isotopic discrimination factors varied with δD of irrigation. The discrimination factors from the deuterium signatures of plants (solid triangles, dotted line), caterpillars (plus ‘+’ signs, barred-dotted line), and moths (open circles, barred line) to that of irrigation water became more negative as the δD value of irrigation water increased. Note that the discrimination factors for plants, caterpillars and moths differed significantly from one another (Tukey's test, p<0.05). The horizontal reference line represents ΔD = 0.

The δD value of extracted soil water closely reflected that of irrigation water (y = −0.98+0.95x, r^2^ = 0.98, N = 20). The value of the intercept of this relationship did not differ significantly from 0 (t_1,9_ = 0.42, P = 0.68), nor did the slope differ significantly from 1 (t_1,9_ = 1.66, P = 0.11, Fig. 1a). δD values of the organic material of plants, caterpillars, and moths were linearly related to the δD values of irrigation water (Fig. 1a). The relationships between the δD value of plant, caterpillar, and moth tissues and that of irrigation water differed in intercept (F_2,62_ = 80.2, p<0.001) but did not differ significantly in slope (F_2,62_ = 0.114, p>0.7). The common slope for plants, caterpillars, and moths was 0.59±0.03 SE, which is significantly lower than 1 (t = 12.6, p<0.0005). Because the slope of the relationship between the organic materials of plants, caterpillars, and moths and the δD value of irrigation water was lower than 1, the ΔD_tissues-irrigation water_ values for plants, caterpillars and moths were negatively correlated with the δD value of irrigation water (Fig. 2).

**Figure 3:**
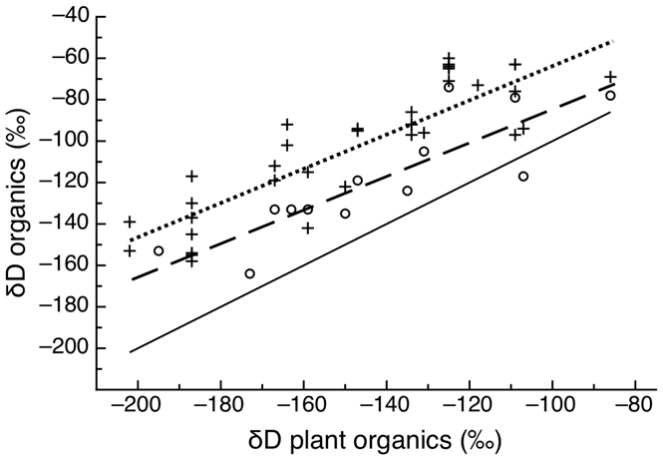
The δD values of moth (open circles, barred line) and caterpillar (plus ‘+’ signs, dotted line) tissues were linearly related to those of plant tissues and had a common slope. Note that moth organic material was significantly depleted in deuterium relative to that of caterpillars.

**Figure 4:**
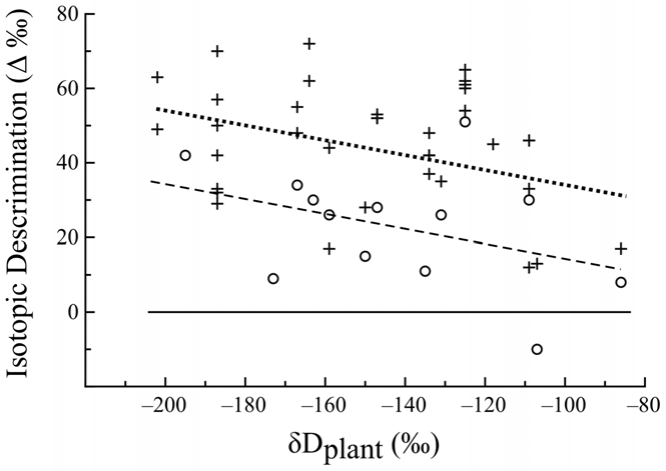
The isotopic discrimination factors between the tissues of caterpillars (plus ‘+’ signs, dotted line) and moths (open circles, barred line) decreased significantly with the δD value of plant tissues according to a shallow common slope. Note that the discrimination factors for plants, caterpillars and moths differed significantly from one another (Tukey's test, p<0.05). The horizontal reference line represents ΔD = 0.

The δD values of both moths and caterpillars were linearly related to the δD value of plant organic matter (Fig. 3). The slope of these linear relationships did not differ among caterpillars and moths (F_1,45_ = 0.16, p = 0.69) and was 0.84 (±0.07, Fig. 3). This slope was significantly lower than 1 (t = 2.3, p = 0.026). Consequently, the discrimination factor between caterpillars and moths and plants decreased significantly with the δD value of plant organic matter (F_1,45_ = 5.16, p = 0.03) with a very shallow common slope (F_1,45_ = 0.16, p = 0.69, slope = −0.16±0.07, Fig. 4). Because the slope was shallow, we can approximate the average Δ_Dconsumer-plant_ values, assuming that plant δD has no effect, as 45‰ (± SD = 3‰) and 23‰ (±SD = 5‰), for caterpillars and moths, respectively. Over our interval of measurements, both caterpillars and moths were significantly enriched in D relative to plants, and caterpillars were enriched relative to moths (F_1, 46_ = 18.6, p<0.001, Fig. 3) by 21‰ (±4.9 SE).

### Hydrogen in water

The δD value of extracted plant water and the body water of caterpillars and moths was positively related to the isotopic composition of irrigation water (Fig. 1b), however, there were significant differences in the slopes of these relationships (F_2,76_ = 10.9, p<0.0001). The relationship between the body water of moths and irrigation water had a significantly lower slope (0.53±0.05 SE) relative to that of both plants and caterpillars (Tukey's test, p<0.05). The slopes and intercepts of the relationships between plant water and caterpillar body water did not differ significantly (Tukey's test p>0.1, common intercept = 43.7‰±2.33 SE, common slope = 0.81±0.03 SE). The value of this slope was significantly lower than 1 (t = 6.3, p<0.01). The δD value of plant water and caterpillar body water was enriched relative to irrigation water and also relative to the tissues of both caterpillars and moths (Fig. 1b).

### Carbon and nitrogen

**Figure 5:**
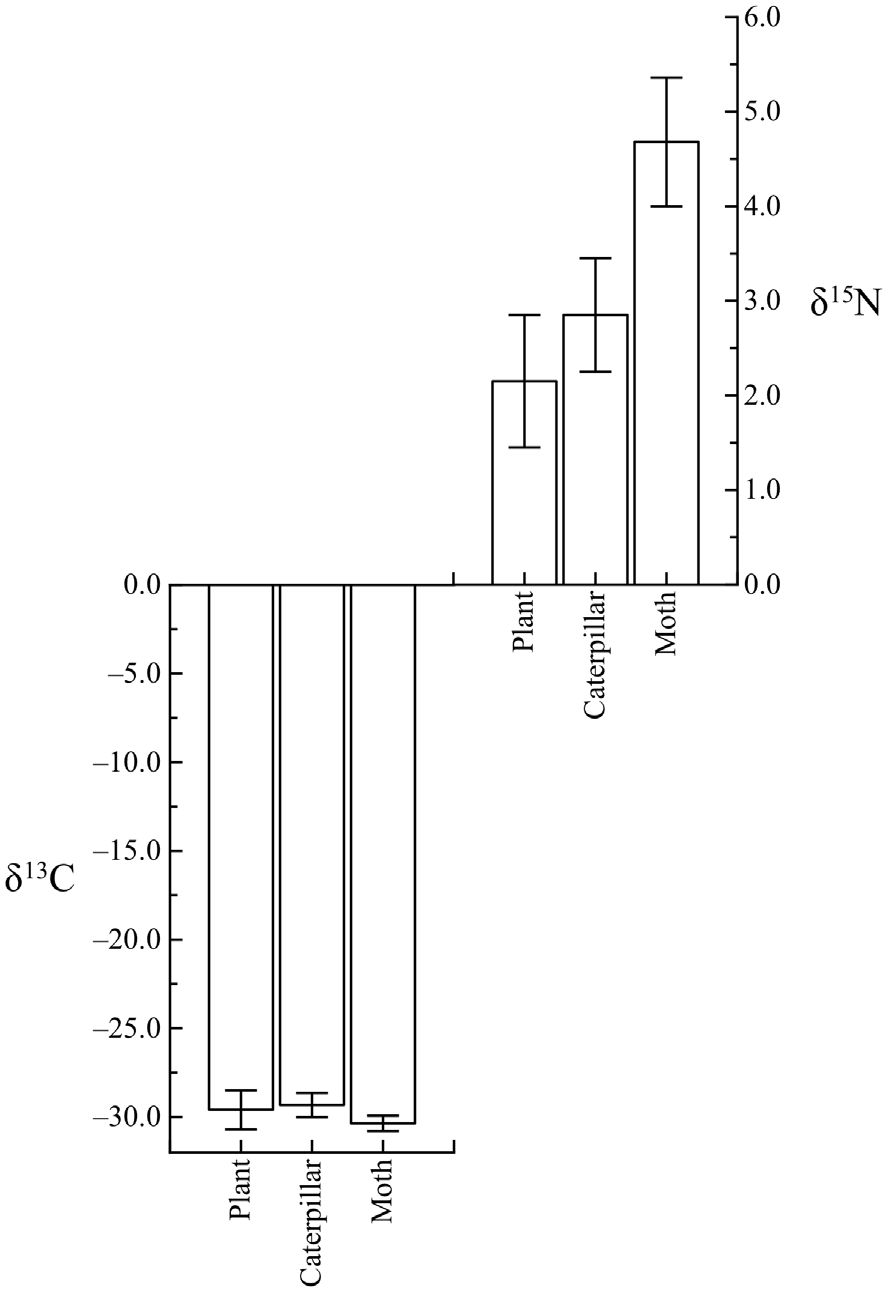
Carbon and nitrogen isotopic values of cabbage plants, caterpillars and moths were influenced by both trophic level and metamorphosis. There were no significant differences in δ^13^C values among plants and caterpillars, but moths were slightly (albeit significantly) depleted in ^13^C relative to both plants and caterpillars. In contrast, δ^15^N values increased significantly from plants to caterpillars to moths. Error bars denote SE.

Because there was no significant effect of water treatment on the δ^13^C and δ^15^N values of cabbage plants (F_3,15_ = 1.2 and F_3,15_ = 0.53, p>0.3 for δ^13^C and δ^15^N, respectively), we pooled all treatments for statistical analysis. There was no significant difference in δ^13^C values between caterpillars and plants (paired t = 1.80, p = 0.7, N = 39, Fig. 5), but moths were slightly, albeit significantly depleted in ^13^C relative to both plants (paired t = 2.15, p = 0.04, N = 28, Table 1) and caterpillars (F_1,69_ = 13.23, p<0.0001). Both caterpillars and moths were significantly enriched in ^15^N relative to plants (paired t>4.8, p<0.0001, Fig. 5, Table 1); moths were significantly enriched in ^15^N relative to caterpillars by 1.5 ‰ (±SE = 0.14, F_1,63_ = 56.3, P<0.001).

**Table 1:** Consumer to plant discrimination factors for caterpillars and moths of *Trichoplusia ni* fed on cabbage.

	Δ^13^C_caterpillar-plant_	Δ^13^C_moth-plant_	Δ^15^N_caterpillar-plant_	Δ^15^N_moth-plant_
	0.3±0.3^NS^	−0.4±0.3^*^	0.7±0.3*	2.4±0.4*
**N**	38	28	31	26

Values are mean ±95% confidence intervals. Values labeled with NS are those for which the confidence interval overlaps with 0, whereas those labeled with * are those for which the 95% confidence interval for the mean does not include 0.

## Discussion

We estimated the discrimination factors at each step along the deuterium trophic pathway from irrigation water to consumer. The magnitude of these trophic discrimination factors varied linearly with the δD value of irrigation. We also documented ‘non-trophic’ discrimination of hydrogen isotopes among different metamorphic stages of cabbage loopers. Note that we use the word “isotopic discrimination” to denote the difference in isotopic composition between a resource and an organism without reference to the many processes that can lead to this difference. In this discussion, we consider the relationship between the δD values of the tissues of plants and insects to those of irrigation water. A likely explanation of why their common slope differs from 1 and why their discrimination factors seem to depend on the isotopic value of irrigation water is also provided. We offer possible causes for the significant enrichment in insect tissues relative to plants and for the differences in isotopic value among larvae and adults. We conclude this discussion by considering several issues raised by our study for stable isotope ecology and specifically for the study of trophic relationships and geographical assignment.

### Tissue to irrigation water discrimination factors: The problem of common garden experiments

Although the δD value of plants, caterpillars and moths varied linearly with the δD value of irrigation water, the slope of these relationships was significantly lower than 1. This deviation from, also reported by Hobson et al. (1999) [10], implies that the extent of hydrogen isotope discrimination for each trophic transfer depends on the δD value of the source water supporting the (greenhouse-fashioned) food web. Specifically, as the δD value of irrigation water increased, the absolute value of the discrimination factor increased (Fig. 2). This is a surprising result. We do not know of any biochemical processes in which enzyme selectivity for a particular isotope is inversely proportional to the amount of the isotope present. This pattern is also uncharacteristic of evaporative fractionation.

Hobson et al. (1999) [10] gave two possible causes for this effect: 1) it might be an idiosyncratic feature of the plant used in the study (milkweeds; *Asclepias* sp.) or 2) discrimination factors might be influenced by atmospheric water vapor. Our results clearly suggest that this effect is not restricted to milkweeds, as it occurs in cabbages as well. Plant physiologists have examined the effect of atmospheric water vapor on the isotopic composition of plant photosynthetic tissues [21]. We used the Craig-Gordon model as modified by [22] to test the hypothesis that stomatal conductance of water facilitates isotopic exchange between leaf water and atmospheric water vapor at the site of transpiration (see [22] for a detailed description of the model). Independent of the atmospheric δD values, we found that the modeled slope of the relationship between δD values of leaf tissue and that of irrigation water had a slope lower than 1, with a magnitude similar to that found in both our study and that by Hobson et al. (1999) [10].

In this model the relationship between the δD value of plant organics and that of δD_irrigation_ is always linear and crucially dependent on relative humidity (rh), but independent of the δD value of atmospheric vapor. As rh tends to 0, the slope of the relationship between δD_organics_ and δD_irrigation_ tends to 0.7. Conversely, if rh tends to 100%, then the slope tends to 1. The observed slope for the relationship between the δD of plant organics and that of irrigation (0.59) is consistent with a low rh (≈20%). The rh in our greenhosue varied from 20–35%. The water vapor in the greenhouse in which the plants were grown was depleted in deuterium relative to all four treatments of irrigation water (ground water and tap water at our study site have a δD value ≈–135‰). Because the isotopic signature of the ambient water vapor was constant, a mixture of irrigation water and ambient water vapor likely caused the δD value of leaf water to deviate from that of irrigation water as the δD value of irrigation decreased. This hypothesis accounts for water at the superficial photosynthetic layers of leaves, but not for water in the whole leaf, including vascular tissue. Theslope of the relationship between δD values of extracted plant water and the δD values of irrigation water was higher (0.83) than that of dry plant tissue (0.59), reflecting a large contribution of soil water.

These data suggest that quantifying discrimination factors between irrigation water and plants grown in a common greenhouse, with irrigation water of various deuterium signatures, is not possible because there are two sources of water hydrogen in the system: irrigation and atmospheric water vapor. We suspect that the δD value of atmospheric water vapor varies linearly with that of local precipitation water in the field, possibly eliminating the ‘common greenhouse effect.’ However, the relative contribution of irrigation water and that atmospheric water vapor likely varies according to relative humidity, temperature, photosynthetic mode and other factors, further confounding the use of discrimination factors between plants and irrigation water determined in field studies.

### Discrimination of deuterium from plants to insects and larvae to adults

We found deuterium enrichments of approximately 45‰ and 23‰ between caterpillars and moths and their food plants, provide new support for the trophic discrimination documented by Birchall et al. [5]. Birchal et al. [5] found highly significant differences (*c.* 90‰) between the δD values of non-exchangeable bone collagen of carnivores and that of herbivores/omnivores of Great Britain, noting that trophic enrichment of deuterium parallels that of ^15^N [1].

Birchall et. al. [5] hypothesized that the incorporation of body water hydrogen during synthesis of non-essential amino acids may be responsible for this enrichment by one, or both, of two mechanisms: 1) body water may provide a deuterium enriched pool of hydrogen available for incorporation during amino acid synthesis, and 2) the incorporation of hydrogen from body water is a discriminating process. The latter mechanism is difficult to assess because caterpillar (and moth) hydrogen can be derived from both dry food matter and ingested water. In support of the first mechanism, the body water of caterpillars was enriched in deuterium relative to the caterpillar's tissues. Because evaporation is a fractionating process that favors light isotopes, the body water of animals tends to be enriched in deuterium (reviewed by [23]). Therefore, the incorporation of hydrogen from an enriched pool into tissues might be a general mechanism that leads to trophic enrichment of deuterium.

We also found that the tissues of adult moths had significantly lower δD values relative to those of caterpillars and hence, the absolute value of the discrimination factor of their tissues relative to plants was smaller (Fig. 4). Like all holometabolous insects, cabbage loopers undergo massive catabolism of existing tissues and synthesis of new tissues during pupation. Moth body water was depleted in deuterium relative to the body water of caterpillars (Fig. 1b). Because lipids have lower δD values relative to proteins [24]–[27], we hypothesize that a significant fraction of the body water in moths is deuterium-depleted metabolic water produced by lipid catabolism during metamorphosis [28]. The incorporation of hydrogen atoms from body water into tissues synthesized during metamorphosis likely explains why moth tissues have significantly lower δD values than caterpillar tissues.

In a similar study, Hobson et al. [10], monarch butterfly larvae and adults (imago) were grown on milkweed host plants. Butterfly wing keratin showed negligible deviation in δD values from that of the host plants, suggesting that trophic enrichment of deuterium had not occurred. We propose several hypotheses for why we found trophic enrichment of deuterium between lepidopteran imago and plants, while Hobson et al. [10] did not. First, Hobson et al. [10] analyzed only wing keratin whereas we analyzed bulk tissue, suggesting that the discrepancy may be related to tissue-specific trophic effects. Second, Hobson et al. [10] considered only imago tissues while we included analyses of both larvae and imago. The trophic enrichment of deuterium from plants to larvae and subsequent depletion from larvae to imago in our study resulted in a net enrichment of deuterium between plants and both caterpillars and imagines. It is possible that trophic enrichment and metamorphic depletion of deuterium also occurred in the monarchs but led to no net discrimination between plants and imago. This would occur if the trophic discrimination and metamorphic discrimination were equal in magnitude, but opposite in sign. Finally, the trophic enrichment may occur in some plant-herbivore systems but not in others.

Our carbon and nitrogen isotope data support observations from previous studies. Although we observed a small moth to plant discrimination (0.4±1.0‰, Fig. 5), the isotopic value of carbon was transferred with little isotopic discrimination across trophic levels [3]. ^15^N is known to bioaccumulate across trophic levels [29]–[31], and indeed, δ^15^N values for caterpillars were greater than that of there food source (plants). We observed that a significant non-trophic ^15^N enrichment took place in the metamorphosis from larvae to adult (Fig. 5) [11].

### Ecological implications

Many areas in animal and plant ecology, ranging from physiological ecology to ecosystem studies, have been transformed by the use of stable isotopes. Many of these applications rely primarily on the use of carbon (δ^13^C) and nitrogen (δ^15^N) isotope values (reviewed by [1]). The study of animal movements, and the study of reciprocal allochthonous and autochthonous flow of materials between terrestrial and aquatic ecosystems [18], often rely on hydrogen isotope (δD) analysis, and to a lesser extent on oxygen isotope (δ^18^O) analyses. The use of δD is complicated because multiple sources of hydrogen from both water and food are combined to biosynthesize tissues. Estimating a discrimination factor when multiple sources are involved is difficult, because discrimination factors can vary from source to source (food, metabolic water, and preformed water) [9], [32], [33] and through different mechanisms on each of these sources. In large rivers, for example, the sources of hydrogen for aquatic consumers include both river water (which might be derived from distant sources), and tissues of autochtonous and allochtonous producers [18]. In other more homogeneous systems, δD has potential as a tracer for identifying water and organic matter sources for consumers, and hence, we can use δD values of tissues to trace animal movements [7], [34]–[41]. However, our results and those of Birchall et al. (2005) [5] indicate that geographic assignment of animal tissues based on δD values may lead to large errors unless the magnitude of and variation in trophic discrimination of hydrogen isotopes is considered. This problem will become even confounding for studies involving omnivorous species that feed at multiple trophic levels. We encourage future investigation of δD trophic discrimination in other food webs [10], [42], [43]. If ecologists can estimate δD trophic discrimination factors for various herbivore-plant and predator-prey relationships, we might be able to enhance the accuracy this method. However, if this trophic discrimination varies greatly among species, they will likely prove useful only for studies of species with known diet composition.
